# Supplementary material for: The usefulness of D-dimer as a predictive marker for mortality in patients with COVID-19 hospitalized during the first wave in Italy
Source: PLoS One. 2022 Jul 22;17(7):e0264106. doi: 10.1371/journal.pone.0264106 (PMC9307169; doi:10.1371/journal.pone.0264106)
Supplement: S1 Table — (DOCX) [file pone.0264106.s001.docx]

**S1 Table. Cumulative incidence of death, per time-period**

| **Time period** | **N** | **Cumulative incidence of death** |
| --- | --- | --- |
| March 6 to September 20 | 507 | 19% (95CI: 16-23) |
| March 6 to March 14 | 35 | 19% (95CI: 7-34) |
| March 14 to March 31 | 266 | 20% (95CI: 15-25) |
| April 1 to April 14 | 127 | 21% (95CI: 14-28) |
| April 14 to April 30 | 52 | 18% (95CI: 9-29) |
| April 30 to September 20 | 27 | 15% (95CI: 4-31) |
