# Supplementary material for: The usefulness of D-dimer as a predictive marker for mortality in patients with COVID-19 hospitalized during the first wave in Italy
Source: PLoS One. 2022 Jul 22;17(7):e0264106. doi: 10.1371/journal.pone.0264106 (PMC9307169; doi:10.1371/journal.pone.0264106)
Supplement: S1 List — (DOCX) [file pone.0264106.s002.docx]

**S2. List of participants in COVID-19 Network working group**

**Fondazione IRCCS Ca’ Granda Ospedale Maggiore Policlinico:**

Scientific Direction: Silvano Bosari, Luigia Scudeller, Giuliana Fusetti, Laura Rusconi, Silvia Dell’Orto.

Department of Transfusion Medicine and Hematology (Biobank): Daniele Prati, Luca Valenti, Silvia Giovannelli, Maria Manunta, Giuseppe Lamorte, Francesca Ferarri.

Infectious Diseases Unit: Andrea Gori, Alessandra Bandera, Antonio Muscatello, Davide Mangioni, Laura Alagna, Giorgio Bozzi, Andrea Lombardi, Riccardo Ungaro, Giuseppe Ancona, Gianluca Zuglian, Matteo Bolis, Nathalie Iannotti, Serena Ludovisi, Agnese Comelli, Giulia Renisi, Simona Biscarini, Valeria Castelli, Emanuele Palomba, Marco Fava, Valeria Fortina, Carlo Alberto Peri, Paola Saltini, Giulia Viero, Teresa Itri, Valentina Ferroni,Valeria Pastore,Roberta Massafra,Arianna Liparoti,Toussaint Muheberimana, Alessandro Giommi, Rosaria Bianco, Rafaela Montalvao De Azevedo, Grazia Eliana Chitani.

Angelo Bianchi Bonomi Hemophilia and Thrombosis Center and Fondazione Luigi Villa: Flora Peyvandi, Roberta Gualtierotti, Barbara Ferrari, Raffaella Rossio, Nadia Boasi, Erica Pagliaro, Costanza Massimo, Michele De Caro, Andrea Giachi.

UOC Internal Medicine, Immunology and Allergology: Nicola Montano, Barbara Vigone, Chiara Bellocchi, Angelica Carandina, Elisa Fiorelli, Valerie Melli, Eleonora Tobaldini.

Respiratory Unit and Cystic Fibrosis Adult Center: Francesco Blasi, Stefano Aliberti, Maura Spotti,Leonardo Terranova, Sofia Misuraca, Alice D’Adda, Silvia Della Fiore, Marta Di Pasquale, Marco Mantero Martina Contarini, Margherita Ori, Letizia Morlacchi, Valeria Rossetti, Andrea Gramegna, Maria Pappalettera, Mirta Cavallini, Agata Buscemi.

Cardiology Unit: Marco Vicenzi, Irena Rota.

Emergency Unit: Giorgio Costantino, Monica Solbiati, Ludovico Furlan, Marta Mancarella, Giulia Colombo, Giorgio Colombo, Alice Fanin, Mariele Passarella.

Acute Internal Medicine: Valter Monzani, Ciro Canetta, Angelo Rovellini, Laura Barbetta, Filippo Billi, Christian Folli, Silvia Accordino.

Rare Diseases Center: Diletta Maira, Cinzia Maria Hu, Irene Motta, Natalia Scaramellini.

General Medicine and Metabolic Diseases: Anna Ludovica Fracanzani, Rosa Lombardi, Annalisa Cespiati.

Geriatric Unit: Matteo Cesari,Tiziano Lucchi,Marco Proietti, Laura Calcaterra, Clara Mandelli, Carlotta Coppola, Arturo Cerizza. Intensive Care Unit: Antonio Maria Pesenti, Giacomo Grasselli, Alessandro Galazzi.

**Istituto di Ricerche Farmacologiche Mario Negri IRCCS**: Alessandro Nobili, Mauro Tettamanti, Igor Monti, Alessia Antonella Galbussera.

**Policlinico G.B. Rossi, Verona:**

UOC di Medicina d’Urgenza: Ernesto Crisafulli, Domenico Girelli, Alessio Maroccia, Daniele Gabbiani, Fabiana Busti, Alice Vianello, Marta Biondan, Filippo Sartori.

**Ospedale San Gerardo, ASST Monza:**

UOC Pneumologia: Paola Faverio, Alberto Pesci, Stefano Zucchetti.

Malattie Infettive: Paolo Bonfanti, Marianna Rossi, Ilaria Beretta, Anna Spolti.

**San Giuseppe Hospital MultiMedica IRCCS and community health, Università degli Studi di Milano**:

UOC Pneumologia: Sergio Harari.

Unità di Pneumologia e terapia Semi-intensiva respiratoria, Servizio di Fisiopatologia Respiratoria ed Emodinamica Polmonare: Davide Elia.

Unità di Pneumologia e terapia Semi-intensiva respiratoria, Servizio di Fisiopatologia Respiratoria ed Emodinamica Polmonare: Roberto Cassandro, Antonella Caminati.

**Ospedale Clinicizzato “SS. Annunziata”**:

Clinica Medica: Francesco Cipollone, Maria Teresa Guagnano, Damiano D’Ardes, Ilaria Rossi, Francesca Vezzani.

**ICS Maugeri Tradate,** **Universita' Insubria:**

Pneumologia Riabilitativa: Antonio Spanevello, Francesca Cherubino, Dina Visca.

**Azienda Ospedaliera Universitaria di Ferrara e Dipartimento di Medicina Traslazionale Università di Ferrara:**

UO Pneumologia: Marco Contoli, Alberto Papi, Luca Morandi, Nicholas Battistini.

**Clinica Polispecialistica San Carlo:**

UO Medicina Interna: Guido Luigi Moreo, Pasqualina Iannuzzi.

UO Oncologia: Daniele Fumagalli.

UO Chirurgia Generale: Sara Leone.
